# Priming Canine Adipose Tissue-Derived Mesenchymal Stem Cells with CBD-Rich Cannabis Extract Modulates Neurotrophic Factors Expression Profile

**DOI:** 10.3390/vetsci12100926

**Published:** 2025-09-24

**Authors:** Vinicius Skau Perino, Lucas Vinícius de Oliveira Ferreira, Beatriz da Costa Kamura, Natielly Dias Chimenes, Alisson Vinícius Gimenes Olbera, Thiago Tourinho Pereira, Aline Márcia Marques Braz, Marjorie de Assis Golim, Márcio de Carvalho, Rogério Martins Amorim

**Affiliations:** 1 Department of Veterinary Clinic, School of Veterinary Medicine and Animal Science, São Paulo State University (UNESP), Botucatu 18618-681, SP, Brazil; v.perino@unesp.br (V.S.P.); lv.ferreira@unesp.br (L.V.d.O.F.); beatriz.kamura@unesp.br (B.d.C.K.); natielly.chimenes@unesp.br (N.D.C.); alisson.olbera@unesp.br (A.V.G.O.); thiago.tourinho@unesp.br (T.T.P.); marcio.carvalho@unesp.br (M.d.C.); 2 Center for Translational Research in Regenerative Medicine, Institute of Biotechnology, São Paulo State University (UNESP), Botucatu 18607-440, SP, Brazil; 3 Laboratory of Applied Biotechnology, Clinical Hospital of the Medical School, São Paulo State University (UNESP), Botucatu 18618-687, SP, Brazil; aline.braz@unesp.br (A.M.M.B.); marjorie.golim@unesp.br (M.d.A.G.)

**Keywords:** phytocannabinoids, cannabidiol, endocannabinoid system, immunomodulation, pre-conditioning

## Abstract

The endocannabinoid system regulates key biological functions such as neuroprotection, pain modulation, inflammation, and immunomodulation. Cannabis-based therapies have gained attention due to the therapeutic potential of their bioactive compounds, particularly phytocannabinoids like cannabidiol (CBD), which exhibit anti-inflammatory, neuroprotective, and immunomodulatory properties. Mesenchymal stem cells (MSCs) are widely studied for their regenerative and immunomodulatory potential. This study evaluated the effects of priming canine adipose tissue-derived MSCs (cAT-MSCs) with a CBD-rich cannabis extract on cell morphology, viability, neurotrophic factor gene expression, and cytokine gene and protein expression. cAT-MSCs (*n* = 5) were primed for 24 h and divided into three groups: Control (C, unprimed), D1 (2.25 µM CBD), and D2 (225 nM CBD). No morphological or viability changes were observed. Gene expression analysis showed that groups D1 and D2 exhibited increased HGF expression. D1 also showed increased IDO and decreased BDNF expression. In contrast, no significant changes were observed in GDNF, IL-10, TNF-α, IFN-γ, or PTGES2. Regarding the cytokine profile, GM-CSF, IL-2, and IL-10 were undetectable. Notably, IL-8 and MCP-1 levels were significantly reduced in D1 compared to the control. These findings suggest that CBD priming modulates key regenerative and inflammatory mediators in cAT-MSCs, supporting its potential application in enhancing the efficacy of cell-based therapies.

## Simple Summary

Mesenchymal stem cells (MSCs) are used in veterinary medicine for their regenerative, immunomodulatory, and anti-inflammatory properties. Compounds from cannabis, especially cannabidiol (CBD), have shown promising anti-inflammatory and healing effects. This study evaluated whether a CBD-rich cannabis extract modulates important regenerative and inflammatory factors in MSCs derived from canine adipose tissue. After priming canine adipose tissue-derived MSCs for 24 h, we found no changes in their morphology or viability. However, the priming with CBD-rich cannabis extract has increased the activity of certain genes linked to tissue repair and reduced the levels of inflammatory cytokines. These results suggest that CBD can influence key factors that help stem cells repair tissue and control inflammation, potentially improving their use in future veterinary therapies.

## 1. Introduction

The therapeutic use of cannabis in both humans and animals has steadily increased over the years [1]. The cannabis plant contains a complex array of bioactive compounds, including the most common phytocannabinoids, Δ9-tetrahydrocannabinol (THC) and cannabidiol (CBD), as well as terpenes, flavonoids, and alkaloids [2,3]. The endocannabinoid system (ECS) is a complex cell-signaling network composed of three main components: endocannabinoids (eCBs), cannabinoid receptor types 1 and 2 (CB1 and CB2), and the enzymes responsible for their synthesis and degradation [4]. Present in all mammals, the ECS plays a crucial role in regulating a wide range of biological and metabolic processes, including neuroprotection, neuroregeneration [5], and immunomodulation [6].

CBD has gained widespread recognition for its therapeutic potential across various medical applications, demonstrating its ability to address a diverse range of health conditions in both humans and animals [7]. It interacts with the ECS modulating key physiological processes such as pain control [8], anxiety and stress reduction, anti-inflammatory responses [9], and epilepsy [10]. Additionally, CBD has been shown to exert neuroprotective effects, supporting neuronal health and potentially aiding neurodegenerative disorders [11], and exhibiting antitumoral properties by modulating cancer cell proliferation and apoptosis, effects observed in both humans and animal models [12].

Mesenchymal stem cells (MSCs) are multipotent progenitor cells capable of differentiating into various specialized cell types. Beyond classical mesodermal lineages, evidence shows MSCs can also differentiate into non-mesodermal lineages, such as neural and endothelial cells, under appropriate stimuli, suggesting a broader phenotypic plasticity [13]. Besides their differentiation potential, MSCs secrete a variety of bioactive factors, including cytokines, growth factors, and extracellular matrix components [14,15]. Moreover, MSCs release extracellular vesicles (EVs), which play a pivotal role in intercellular communication [16]. These EVs are enriched with proteins, lipids, mRNAs, and regulatory non-coding RNAs like microRNAs, significantly contributing to the immunomodulatory, anti-inflammatory, and regenerative properties attributed to MSC-based therapies, which aid tissue repair and regeneration [17]. MSCs are also characterized by their immunomodulatory properties, enabling them to modulate immune responses and reduce inflammation, making them valuable in treating immune-mediated disorders [18,19]. Furthermore, MSCs exhibit regenerative capabilities that facilitate tissue healing and repair, promoting the restoration of damaged tissues [20].

Priming MSCs refers to the process of exposing these cells to various environmental factors, including growth factors [21], cytokines [22], bioactive compounds [23], physical stimuli [24], and 3D culture conditions [25]. This process aims to enhance their therapeutic potential by modulating cell behavior and characteristics prior to clinical application [26]. MSCs primed with substances like phytocannabinoids show increased efficacy in treating pathological conditions, including Alzheimer’s disease [27], improved differentiation and viability [28], enhanced migration capacity [29], and more effective tissue repair [30]. Priming with cannabis-derived bioactive compounds often amplifies MSCs’ regenerative and immunomodulatory properties, making them more effective for applications such as neurodegenerative diseases, tissue engineering, and injury repair [31].

In this in vitro study, we evaluated cellular morphology, viability, and gene expression of neurotrophic factors (brain-derived neurotrophic factor—BDNF, glial cell line-derived neurotrophic factor—GDNF, hepatocyte growth factor—HGF), cytokines (Interleukin-10—IL-10, tumor necrosis factor-alpha—TNF-α, Interferon-gamma—IFN-γ), and other immunomodulatory genes (indoleamine 2,3-dioxygenase—IDO, prostaglandin E2 Synthase 2—PTGES2), as well as cytokine secretion profiles (Granulocyte-Macrophage Colony-Stimulating Factor—GM-CSF, Interleukin-2—IL-2, Interleukin-8—IL-8, IL-10, monocyte chemoattractant protein-1—MCP-1), following priming of cAT-MSCs with a CBD-rich cannabis extract at 2.25 µM and 225 nM for 24 h. The purpose of this study was to evaluate the potential effects of priming canine adipose tissue-derived MSCs (cAT-MSCs) with a CBD-rich cannabis extract, aiming to advance our understanding of phytocannabinoid interactions with these cells and assess whether this priming strategy can enhance their immunomodulatory and neuroregenerative properties.

## 2. Materials and Methods

### 2.1. Animal Ethics Committee

This study was conducted in accordance with the Ethical Principles in Animal Experimentation and was approved by the Ethics Committee on the Use of Animals (CEUA) of the School of Veterinary Medicine and Animal Science, São Paulo State University (UNESP), under protocol number 0171/2021.

### 2.2. Experimental Design

**Figure 1:**
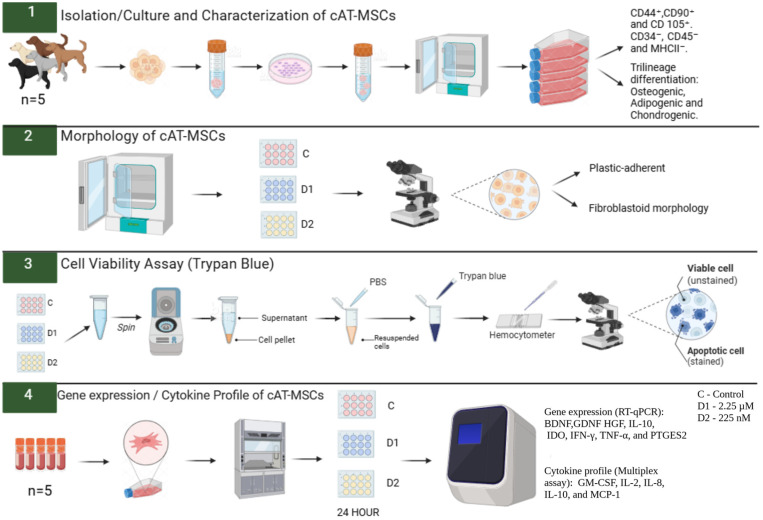
Experimental design. Canine adipose tissue-derived mesenchymal stem cells (cAT-MSCs) were thawed and cultured in 75 cm^2^ flasks. Upon reaching 80% confluence, the cells were plated in 24-well plates and primed with a Cannabidiol (CBD)-rich cannabis extract at two concentrations (2.25 µM and 225 nM). After 24 h of priming, cell viability was assessed using the 0.4% Trypan Blue test, and morphology was evaluated using an inverted microscope. Subsequently, the expression of neurotrophic factors, cytokines, and immunomodulatory genes was analyzed by RT-qPCR, and cytokine levels in the conditioned medium were quantified using a Multiplex Assay. PBS: phosphate-buffered saline. C: Control. D1: dose 1 (2.25 µM). D2: dose 2 (225 nM). BDNF: brain-derived neurotrophic factor. GDNF: glial cell line-derived neurotrophic factor. HGF: hepatocyte growth factor. IL-10: interleukin-10. IDO: Indoleamine 2,3-dioxygenase. IFN-γ: interferon-gamma. TNF-α: tumor necrosis factor-alpha. PTGES2: prostaglandin E2 synthase 2. GM-CSF: granulocyte-macrophage colony-stimulating factor. IL-2: interleukin-2. IL-8: Interleukin-8. MCP-1: monocyte chemoattractant protein-1. Created in BioRender. Amorim, R. (2025) https://BioRender.com/2q4ca6a, accessed on 19 September 2025.

The experiment was conducted in duplicate, and the cells were primed for 24 h. The cAT-MSCs were divided into three groups: Control (C)—cells unprimed and cultured in standard Dulbecco’s Modified Eagle’s Medium (DMEM); Dose 1 (D1)—cells primed with a CBD-rich cannabis extract at 2.25 µM; and Dose 2 (D2)—cells primed with a CBD-rich cannabis extract at 225 nM. These concentrations were selected based on previous studies demonstrating that CBD in the low nanomolar to low micromolar range can modulate MSC viability, migration, and paracrine activity without inducing cytotoxic effects [32,33]. After adhering to the plate, the cAT-MSCs were primed with the CBD-rich cannabis extract for 24 h. Following priming, cellular morphology was evaluated, the supernatant was removed and frozen, and cell viability was assessed using trypan blue. Subsequently, the cells were collected with TRIzol™ (Invitrogen^TM^, Thermo Fisher Scientific, Waltham, MA, USA) and cryopreserved at −80 °C until subsequent analysis of neurotrophic factor and cytokine gene expression by real-time quantitative polymerase chain reaction (RT-qPCR). Cytokine levels in the conditioned medium were quantified using a Multiplex Assay (Figure 1).

### 2.3. Isolation, Cultivation, and Characterization of Canine Adipose-Derived Mesenchymal Stem Cells

cAT-MSCs (P3), derived from adipose tissue (*n* = 5), were obtained from the subcutaneous fat of healthy mixed-breed female dogs, aged 6 to 60 months, undergoing routine abdominal surgery. All donor animals were admitted to the Veterinary Teaching Hospital, School of Veterinary Medicine and Animal Science, São Paulo State University (UNESP), Botucatu, SP. These cells were isolated, cultured, and characterized by our research group. Briefly, 1 g of adipose tissue was minced and digested with 0.1% type I collagenase at 37 °C for 30 min.

Enzymatic digestion was terminated using 90% DMEM/F12 supplemented with 10% fetal bovine serum (FBS) (both from Nova Biotecnologia, Cotia, SP, Brazil). After centrifugation, the medium was filtered to remove any remaining lipid layer, and the cell pellet was washed before being plated onto tissue culture flasks in standard medium for incubation. Once the cells reached approximately 80% confluence, cAT-MSCs were cryopreserved at passages P1–P3 using a cryopreservation medium containing FBS and 10% dimethyl sulfoxide (DMSO) (Synth, Diadema, SP, Brazil) and later thawed for subsequent experiments.

cAT-MSCs were evaluated for surface cluster of differentiation (CD) antigen profile using flow cytometry (FACSCalibur, Becton Dickinson Company Franklin Lakes, NJ, USA). Their ability to differentiate into osteogenic, adipogenic, and chondrogenic lineages was confirmed in vitro under specific induction conditions. The cells had been previously characterized based on adherence to plastic, fibroblast-like morphology, immunophenotypic profile, and multipotent differentiation potential. The cultures (*n* = 5) adhered to the plastic surface of the culture flasks and exhibited a typical fibroblast-like morphology, reaching 80–90% confluence within seven days after initial plating. In passages P1 to P3, cultures reached approximately 80% confluence within 5 to 6 days. Immunophenotypic analysis by flow cytometry showed high expression of the mesenchymal markers CD29 (99.62%) and CD44 (99.19%). In contrast, expression of the hematopoietic markers CD45 (2.04%), CD14 (1.71%), and CD34 (1.59%), as well as the MHC class II (1.71%), was negligible. These findings are in accordance with previous studies [34,35].

For the experiment, cAT-MSCs were thawed, plated in 75 cm^2^ culture flasks (Kasvi, Pinhais, PR, Brazil), and expanded in standard culture medium, consisting of 90% DMEM/F12, 10% FBS, 1% penicillin-streptomycin, and 0.5% amphotericin B (all from Nova Biotecnologia, Cotia, SP, Brazil). Once the cells reached 70–80% confluence, cAT-MSCs were seeded at a density of 1 × 10^5^ cells per well in 24-well plates (Kasvi, Pinhais, PR, Brazil), using a total volume of 500 µL of culture medium per well. The procedure was performed in duplicate.

### 2.4. CBD-Rich Cannabis Extract

The cannabis extract used in this study was obtained from Maria Flor Associação Canábica (Marília, SP, Brazil). The full-spectrum extract was analyzed by High-Performance Liquid Chromatography (HPLC) to determine the concentrations of CBD and THC, showing 28.12% CBD and 0.8% THC per gram of extract (DALL—Analytical and Business Solutions, Boa Vista, Curitiba, Brazil). Subsequently, the cannabis extract was diluted in DMSO at a 1:1 ratio, filtered, and then further diluted in DMEM to achieve concentrations of 2.25 µM and 225 nM, which were utilized in this study.

### 2.5. Morphological Evaluation

The morphology of cAT-MSCs was evaluated 24 h after priming in the Control group (C), D1 group (2.25 µM CBD-rich cannabis extract), and D2 group (225 nM CBD-rich cannabis extract), using an inverted microscope (LEICA DMIRB, Wetzlar, Germany) to obtain photomicrographs.

### 2.6. Cell Viability Assessment

After 24 h of priming, cell viability of the C group, D1 group (2.25 µM CBD-rich cannabis extract), and D2 group (225 nM CBD-rich cannabis extract) was assessed using 0.4% Trypan Blue. cAT-MSCs were collected, mixed with Trypan Blue solution at a 1:1 ratio, and loaded into a Neubauer counting chamber. Cell viability was expressed as a percentage using the formula: Viability (%) = (Number of viable cells × 100) ÷ Total number of cells (viable + non-viable).

### 2.7. cAT-MSCs Cytokines and Neurotrophic Factors Gene Expression

After 24 h of priming, cell lysis was performed in the C, D1, and D2 groups using TRIzol reagent (Invitrogen^TM^, Thermo Fisher Scientific, Waltham, MA, USA). RNA extraction was also conducted using the same reagent, following the manufacturer’s instructions. RNA concentration and purity were assessed using a NanoDrop 2000 spectrophotometer (Thermo Fisher Scientific, Wilmington, DE, USA), based on the 260/280 nm and 260/230 nm absorbance ratios. cDNA synthesis was carried out using the High-Capacity cDNA Reverse Transcription Kit reagents (Applied Biosystems™, Life Technologies Corporation, Carlsbad, CA, USA), following the manufacturer’s instructions. Reverse transcription was performed using a Veriti™ 96-Well Thermal Cycler (Applied Biosystems™, Thermo Fisher Scientific, Waltham, MA, USA). For thermocycling, the following conditions were used: 10 min at 25 °C, 12 min at 37 °C, and 5 min at 85 °C. The resulting cDNA was diluted in RNA-free water to a final volume of 110 μL and stored at 80 °C until further analysis.

PCR reactions were performed in duplicate utilizing cDNA generated with PowerUp^TM^ SYBR^TM^ Green Master Mix (Applied Biosystems^TM^, Thermo Fisher Scientific, Waltham, MA, USA), RNA-free water, and canine-specific primers (Thermo Fisher Scientific, São Paulo, SP, Brazil), designed with Primer Express^TM^ Software v3.0.1 (Applied Biosystems^TM^, Thermo Fisher Scientific, Waltham, MA, USA). Each oligonucleotide primer was individually designed based on sequences obtained from the GeneBank^®^ database (NIH, Genetic Sequence Database) (Table 1).

Eight canine target genes were analyzed: *BDNF*, *GDNF*, *HGF*, *IL-10*, *IDO*, *PTGES2*, *IFN-γ*, and *TNF-α*. Three endogenous canine genes were used: hypoxanthine–guanine phosphoribosyl transferase (*HPRT*); glyceraldehyde-3-phosphate dehydrogenase (*GAPDH*); ribosomal protein S5 (*RPS5*); and ribosomal protein S19 (*RPS19*). The relative quantification of the target genes was performed using the ΔΔCt method [36].

### 2.8. Cytokine Profile in Conditioned Medium

Cytokine levels were assessed in the conditioned medium of the C, D1, and D2 groups 24 h after priming, using the Luminex^®^ Multiplex Assay panel (CCYTOMAG-90K, Merck KGaA, Darmstadt, Germany). GM-CSF, IL-2, IL-8, IL-10, and MCP-1 were quantified according to the manufacturer’s instructions, using the MAGPIX^®^ 200™ analyzer (Merck KGaA, Darmstadt, Germany). Fluorescence emitted by the analytes was measured using the Luminex xPONENT^®^ 4.3 software. Standard curves were generated, and data were analyzed using MILLIPLEX^®^ Analyst 5.1 software.

### 2.9. Statistical Analysis

Cell viability data were analyzed using one-way analysis of variance (ANOVA). Variables related to relative gene expression and cytokine levels did not follow a normal distribution; therefore, group comparisons were performed using the non-parametric Kruskal–Wallis test. When significant differences were detected, median values were compared using Dunn’s post hoc test. Statistical significance was set at *p* < 0.05 (*). All statistical analyses and graph generation were performed using GraphPad Prism version 9.5 for Windows (GraphPad Software, San Diego, CA, USA).

## 3. Results

### 3.1. Morphology

**Figure 2:**
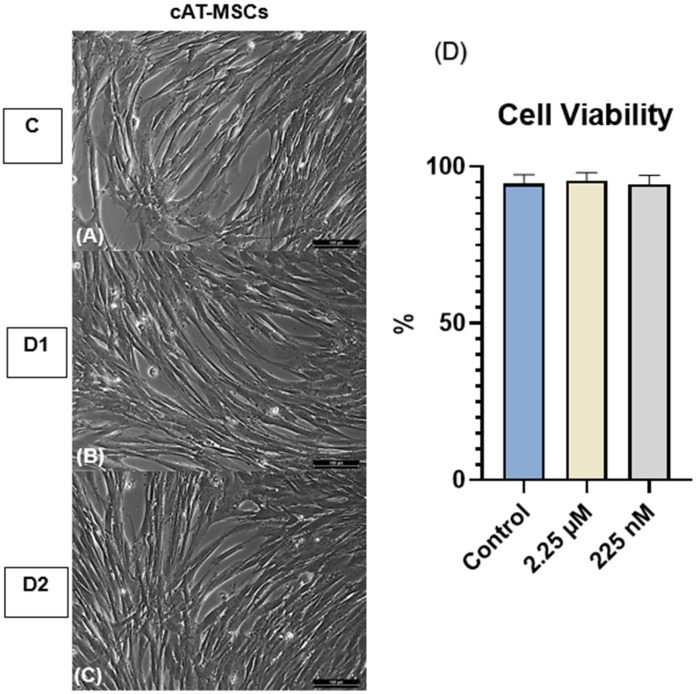
Morphology and cell viability of canine adipose tissue-derived mesenchymal stem cells (cAT-MSCs). (**A**) Control group (C; unprimed), (**B**) D1, and (**C**) D2 cells primed at concentrations of 2.25 µM and 225 nM, respectively, showing fibroblast-like morphology with no observable alterations compared to the control. Magnification, ×200. Scale bar = 100 µm. (**D**) Cell viability of unprimed and CBD-primed cAT-MSCs, with no significant differences observed (*p* > 0.05). Data are presented as mean ± standard deviation. One-way analysis of variance (ANOVA).

No morphological alterations were observed in cAT-MSCs primed with the CBD-rich cannabis extract across the C, D1, and D2 groups (Figure 2).

### 3.2. Cell Viability

Cell viability was assessed using the 0.4% Trypan Blue exclusion assay. The mean viability rates were 94.6% in the control group (C), 95.6% in the D1 group, and 94.4% in the D2 group. No statistically significant differences were observed among the groups (Figure 2).

### 3.3. Gene Expression of Cytokines and Neurotrophic Factors

**Figure 3:**
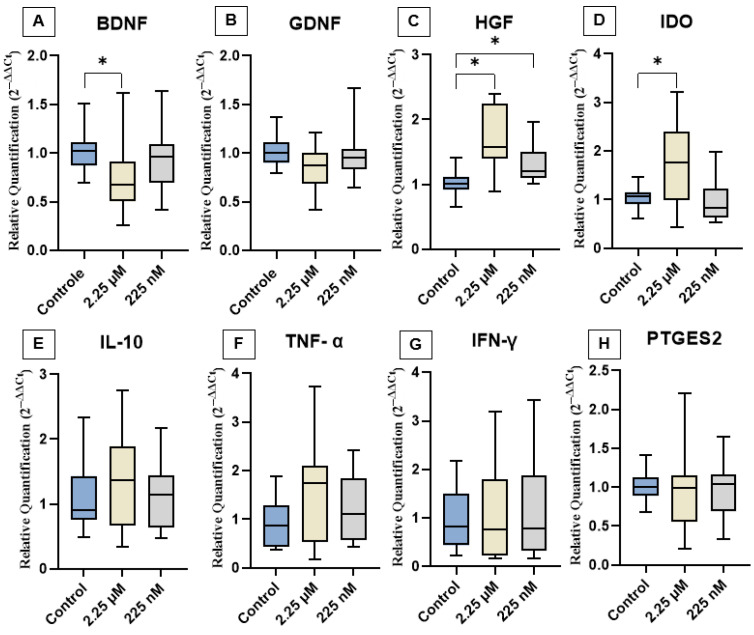
Relative expression of neurotrophic factors, immunomodulatory genes, and cytokines among the experimental groups. (**A**) *BDNF*, (**B**) *GDNF*, (**C**) *HGF*, (**D**) *IDO*, (**E**) *IL-10*, (**F**) *TNF-α*, (**G**) *IFN-γ*, and (**H**) *PTGES2*. Data are represented as medians, interquartile ranges, and minimum and maximum values (* *p* < 0.05). Kruskal–Wallis and Dunn’s tests. BDNF: Brain-derived neurotrophic factor; GDNF: Glial cell line-derived neurotrophic factor; HGF: Hepatocyte growth factor; IDO: Indoleamine 2,3-dioxygenase; IL-10: Interleukin-10; TNF-α: Tumor necrosis factor-alpha; IFN-γ: Interferon-gamma; PTGES2: Prostaglandin E synthase 2.

The effects of priming cAT-MSCs with a CBD-rich cannabis extract on the expression of neurotrophic factors (BDNF, GDNF, and HGF), cytokines (IL-10, TNF-α, and IFN-γ), and immunomodulatory genes (IDO and PTGES2) are shown in Figure 3. Gene expression analysis revealed that both D1 and D2 groups exhibited a significant increase in HGF expression compared to the control. Additionally, D1 showed upregulation of IDO and downregulation of BDNF. In contrast, no significant differences were observed in the expression levels of GDNF, IL-10, TNF-α, IFN-γ, or PTGES2 among the groups.

### 3.4. Cytokine Profile

**Figure 4:**
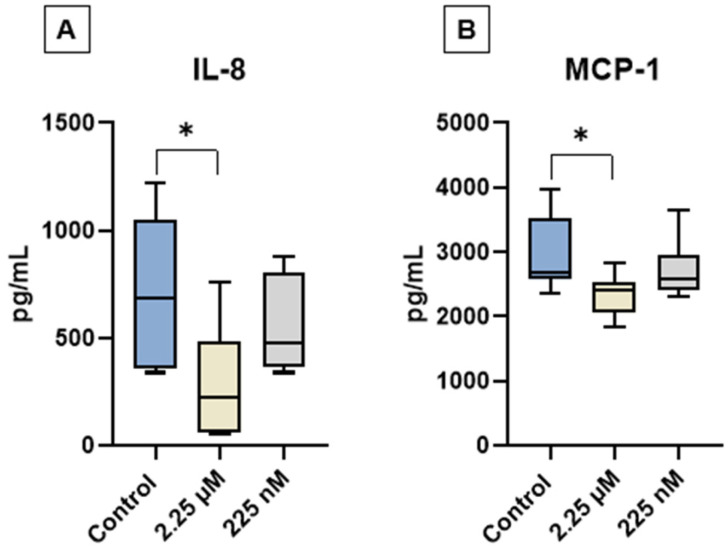
Quantification of interleukin-8 (IL-8) and monocyte chemoattractant protein-1 (MCP-1) in the conditioned medium of canine adipose tissue-derived mesenchymal stem cells (cAT-MSCs) after priming with a CBD-rich cannabis extract. (**A**) IL-8 and (**B**) MCP-1 levels. Data are represented as medians, interquartile ranges, and minimum and maximum values (* *p* < 0.05). Kruskal–Wallis and Dunn’s tests.

The levels of GM-CSF, IL-2, IL-8, IL-10, and MCP-1 were quantified in the conditioned medium of cAT-MSC cultures from the experimental groups C, D1, and D2 using a multiplex assay. GM-CSF, IL-2, and IL-10 were not detected in any of the samples. In contrast, IL-8 and MCP-1 were consistently detected across all groups. Notably, a significant reduction in IL-8 and MCP-1 levels was observed in the D1 group compared to the control (Figure 4).

## 4. Discussion

Although the regenerative and immunomodulatory potential of MSCs is well recognized, the success of MSC-based therapies remains limited due to several interfering factors, such as sensitivity to adverse microenvironments and variability in immunomodulatory responses [37].

In the present study, no morphological changes were observed in cAT-MSCs at the tested concentrations, and the cells retained their characteristic fibroblast-like shape. Additionally, cell viability was not affected. These findings suggest that priming with a CBD-rich Cannabis extract, at the tested concentrations, does not compromise cell integrity.

Priming cAT-MSCs with the CBD-rich extract significantly modulated specific neurotrophic factors, particularly increasing HGF expression and decreasing BDNF levels, indicating a differential response to cannabinoid exposure. Several in vitro studies have demonstrated that CBD interacts with MSCs, enhancing migration, regenerative capacity, and antioxidant activity at concentrations ranging from 10 nM to 15 µM [38,39]. Furthermore, evidence suggests that priming AT-MSCs and bone marrow-derived MSCs with phytocannabinoids, especially at lower doses, can potentiate their regenerative effects [29].

The significant upregulation of HGF suggests that the CBD may stimulate paracrine signaling pathways via cannabinoid-responsive receptors expressed on MSCs, such as CB2, transient receptor potential vanilloid 1 (TRPV1), or peroxisome proliferator-activated receptor gamma (PPARγ), which are involved in cell survival, trophic factor secretion, and immunomodulation [40,41]. The observed modulation indicates that CBD functions not only as an anti-inflammatory agent but also as a bioactive modulator capable of enhancing specific functional outputs of MSCs, thereby reinforcing its potential in regenerative and neurotherapeutic applications [42,43]. Its upregulation may be positively influenced by CBD, likely due to CBD’s ability to modulate cell proliferation [44], promote differentiation [45], exert anti-inflammatory effects [46], and support tissue regeneration [47].

Additionally, CBD may reduce liver fibrosis, possibly through activation of CB2 receptors, which are associated with anti-fibrotic effects [48]. Likewise, HGF plays a key role in liver regeneration [49], and there is growing evidence suggesting that CBD may offer therapeutic benefits in the context of liver disease.

Although CBD is known for its neuroprotective properties, we observed a reduction in BDNF gene expression, suggesting a complex regulatory mechanism that may be influenced by factors such as dosage, duration of exposure, or feedback inhibition—issues that warrant further investigation. Previous studies have shown that CBD can modulate BDNF expression through its interaction with the ECS, which plays a critical role in regulating neuroplasticity and neuronal survival [50,51]. CBD also influences the ECS by inhibiting the degradation of the endocannabinoid anandamide (AEA), thereby enhancing the activation of cannabinoid receptors (CB1 and CB2), which may contribute to its neuroprotective effects [52,53]. Moreover, CBD positively regulates the PI3K/Akt/mTOR signaling pathway, decreases pro-inflammatory mediators such as IFN-γ and IL-17, increases PPARγ activity, and promotes neuronal survival through inhibition of the MAPK pathway [54,55].

Priming of cAT-MSCs with a CBD-rich cannabis extract led to a significant increase in IDO gene expression at the 2.25 µM concentration. IDO is a key immunoregulatory enzyme involved in the catabolism of tryptophan via the kynurenine pathway, playing a critical role in promoting immune tolerance, suppressing T cell proliferation, and modulating local inflammatory responses [56,57]. Its expression is commonly upregulated in response to inflammatory stimuli, particularly in the presence of IFN-γ. Although CBD is widely recognized for its anti-inflammatory and immunomodulatory properties, IDO expression in MSCs is generally inducible rather than constitutive and strongly depends on exposure to inflammatory mediators [58]. Therefore, the increased IDO expression observed in our study may suggest that CBD is capable of partially mimicking or enhancing immune-regulatory pathways even in the absence of an overtly pro-inflammatory environment, potentially enhancing the therapeutic capacity of MSCs.

Emerging evidence suggests that CBD can modulate immune responses and cytokine secretion [59] through indirect regulation of key signaling pathways, including NF-κB and JAK/STAT, which are known to influence IDO expression [60]. While direct mechanisms linking CBD to IDO transcription or enzymatic activity remain incompletely understood, our findings support the hypothesis that CBD may enhance IDO gene expression under specific conditions, as observed with the 2.25 µM concentration. Previous studies have indicated that CBD’s effects on IDO can be highly variable and dependent on the cellular context [61,62]; however, our results add to a growing body of data showing that cannabinoid-based priming can induce relevant functional changes in MSCs, particularly through the modulation of factors such as IDO, HGF, IL-8, and MCP-1, enhancing their therapeutic potential.

In our study, IL-10 gene expression remained unchanged following priming of cAT-MSCs with CBD-rich cannabis extract at both tested concentrations. Despite this finding, there is evidence supporting CBD’s ability to upregulate IL-10 production, particularly in models characterized by immune activation or ongoing inflammation [63,64]. IL-10 is a pivotal anti-inflammatory cytokine, often described as a key mediator of immune tolerance. It acts by suppressing the expression of pro-inflammatory cytokines [65], antigen presentation, and the activation of T cells, monocytes, and macrophages [66]. However, in our in vitro model, no pro-inflammatory stimulus was applied, and CBD alone may not have been sufficient to trigger IL-10 transcriptional activation in cAT-MSCs. This may have occurred because IL-10 upregulation by CBD often depends on the presence of inflammatory signaling, such as elevated NF-κB or STAT3 activity [67]. Our data thus suggest that CBD’s capacity to induce IL-10 may be context-dependent, requiring a primed or activated immune state to manifest. These results support the broader understanding that MSCs respond dynamically to environmental cues and that their secretory and transcriptional profiles, including IL-10 expression, are shaped by the surrounding microenvironment.

Priming cAT-MSCs with a CBD-rich cannabis extract did not result in significant changes in the gene expression of the pro-inflammatory cytokines TNF-α and IFN-γ. These cytokines play central roles in immune regulation: TNF-α is primarily produced by activated macrophages and regulates inflammation, apoptosis, and immune signaling, while IFN-γ is crucial for antiviral defense and broader immune activation [68,69]. Although CBD is widely recognized for its anti-inflammatory effects, including the suppression of pro-inflammatory cytokine production and inhibition of pathways such as NF-κB [70], our findings suggest that, under the conditions tested, CBD priming does not significantly alter the basal gene expression of TNF-α and IFN-γ in cAT-MSCs. This observation may be related to the fact that these genes were not highly expressed in non-inflammatory conditions or that CBD’s regulatory effects on these cytokines may require an inflammatory stimulus to be evident.

Previous studies have demonstrated that CBD can inhibit NF-κB activation and reduce the expression of TNF-α and IFN-γ, especially in the presence of immune stimulation or injury [70,71]. However, in a steady-state or naïve MSC culture environment, the expression levels of these cytokines may be too low for CBD to exert measurable downregulatory effects. Moreover, the effect of CBD on inflammatory mediators is highly context-dependent, varying according to the cell type, the presence of external stimuli, the dose, and the duration of exposure. These findings suggest that the immunomodulatory action of CBD may be more pronounced under inflammatory or stress-induced conditions.

Regarding PTGES2, no significant change in gene expression was observed after CBD priming. While this might suggest a limited direct effect under the tested conditions, it is essential to consider the biological role of PTGES2 and the known effects of CBD on inflammatory signaling. PTGES2 is a key enzyme in the biosynthetic pathway of prostaglandin E2 (PGE2), a lipid mediator involved in regulating inflammation, immune responses, and nociception, is synthesized through a cascade that involves cyclooxygenase enzymes (COX-1/COX-2) followed by prostaglandin E synthases like PTGES2 [72,73]. Evidence suggests that CBD exerts anti-inflammatory effects, in part, by inhibiting COX-2 activity and downstream PGE2 synthesis, although PTGES2 is not always the primary target; the inhibition of upstream enzymes such as COX-2 may indirectly downregulate PGE2 production and influence PTGES2 expression or activity [74,75,76]. Nonetheless, the absence of a significant change in PTGES2 expression may be explained by the lack of a pro-inflammatory stimulus in the in vitro environment. MSCs under basal (non-inflammatory) conditions may not actively express elevated levels of PTGES2, and thus, the impact of CBD might be limited or only become apparent when cells are primed with inflammatory cues, such as TNF-α or lipopolysaccharide (LPS) [77]. Taken together, our findings suggest that CBD priming alone is insufficient to alter PTGES2 transcription in unstimulated cAT-MSCs; however, this does not preclude the functional modulation of the prostaglandin pathway under inflammatory conditions. Future studies incorporating inflammatory stimuli may better reveal the regulatory effects of CBD on PTGES2 and prostaglandin signaling in MSCs.

Although the expression levels of GDNF and all evaluated cytokines did not reach statistical significance compared to controls, some of these markers showed trends toward modulation. These trends, while not statistically conclusive, may indicate subtle shifts in the immunoregulatory and neurotrophic profiles of cAT-MSCs following cannabinoid exposure. Such findings suggest potential biological relevance and underscore the need for further studies with larger sample sizes, extended observation periods, or different extract concentrations to better characterize the molecular mechanisms involved. The absence of significant changes in classic anti- and pro-inflammatory cytokines may also imply that the primary effect of CBD in this context is directed more toward trophic support rather than overt immunosuppression.

The analysis of the secretome of cAT-MSCs following priming with a CBD-rich cannabis extract revealed selective modulation of cytokine secretion. Notably, GM-CSF, IL-2, and IL-10 were not detected in any of the experimental groups, suggesting that these soluble factors are either minimally secreted under basal culture conditions or are not influenced by CBD priming at the tested concentrations. This finding aligns with previous reports indicating that MSCs secrete these cytokines at low or variable levels in vitro, unless stimulated by inflammatory cues or specific microenvironmental conditions [78,79].

In contrast, IL-8 and MCP-1 were robustly expressed in all groups, confirming their role as key components of the cAT-MSC secretome. Both cytokines are well-known for their involvement in immunomodulation, chemoattraction of immune cells, and paracrine signaling [80]. Interestingly, the secretion of both IL-8 and MCP-1 was notably reduced in the group primed with 2.25 µM of CBD (D1) compared to the control and the secretion of both IL-8 and MCP-1 was notably reduced in the group primed with 2.25 µM of CBD (D1) compared to the control. This finding is consistent with the well-known anti-inflammatory properties of CBD, suggesting that priming at this concentration may reduce the release of pro-inflammatory chemokines and promote a more immunomodulatory secretory profile. Taken together, these findings suggest that CBD priming does not broadly suppress the cAT-MSC secretome, but rather selectively modulates key cytokines involved in inflammation and immune cell recruitment.

This study presents some limitations that should be considered when interpreting the lack of protein-level analysis of additional cytokines and neurotrophic factors. The experimental design relied on basal in vitro conditions, without the introduction of inflammatory stimuli, which may have limited the activation of key immunomodulatory pathways typically responsive to cannabinoid exposure. Additionally, the use of two concentrations of the CBD-rich cannabis extract and a single exposure time may not fully capture the dose- or time-dependent dynamics of MSC responses. Future studies incorporating inflammatory preconditioning, a broader range of extract concentrations, and functional assays will be essential to better define the therapeutic potential of CBD-primed MSCs.

## 5. Conclusions

Priming cAT-MSCs with a higher concentration of a CBD-rich extract (D1—2.25 µM) significantly increased HGF and IDO gene expression, decreased BDNF expression, and reduced IL-8 and MCP-1 protein levels compared to the control. In contrast, the lower dose (D2—225 nM) led to increased HGF gene expression only. These results suggest that CBD-rich extract priming, especially at the higher concentration of 2.25 µM, has preliminary modulatory effects on key regenerative and immunomodulatory mediators in cAT-MSCs in vitro, and further studies using in vivo models are needed to assess their potential biological relevance.

## Figures and Tables

**Table 1:** Primer sequences used in RT-qPCR reactions.

Gene	Forward	Reverse
*BDNF*	GTGTCGAAAGGCCAACTGAAG	CGTGTAACCCATGGGATTGC
*GDNF*	GGTTTGCTACAGCCAGCAGTT	CGCACCATGTTCAAAATCCA
*HGF*	ATGGTTCTTGGCGTCATTGTT	AATGCCAGGACGATTTGGAA
*IL-10*	CCCAGGATGGCAACTCTTCTC	CGGGATGGTATTTTGCAGATC
*IDO*	TGTGGACCCAAGCACGTTTT	AGTTGCCTTTCCAACCAGACA
*PTGES2*	GCCTGCAGCTGACCCTGTA	CACGGACCTTGCTGCAGAA
*IFN-γ*	TCTCACCAAGATCCAACC TAAGG	TGCGGCCTCGAAACAGA
*TNF-α*	TGGAATCATTGCCCTGTAAGG	TGATCAAAGGGTCGGTTTGG
*HPRT*	CGGCTTGCTCGAGATGTGAT	GCACACAGAGGGCTATGT
*RPS5*	GAGGCCTCAGGCTGTCGAT	AGCCAAATGGCCTGATTCAC
*RPS19*	GGGTCCTCCAAGCCCTAGAG	CGGCCCCCATCTTGGT

*BDNF*: Brain-derived neurotrophic factor; *GDNF*: Glial cell line-derived neurotrophic factor; *HGF*: Hepatocyte growth factor; *IL-10*: Interleukin-10; *IDO*: Indoleamine 2,3-dioxygenase; *PTGES2*: Prostaglandin E synthase 2; *IFN-γ*: Interferon-gamma; *TNF-α*: Tumor necrosis factor-alpha; *HPRT*: Hypoxanthine phosphoribosyltransferase; *RPS5*: Ribosomal protein S5; *RPS19*: Ribosomal protein S19.

## Data Availability

The original contributions presented in the study are included in the article, further inquiries can be directed to the corresponding author.

## Abbreviations

The following abbreviations are used in this manuscript:

AEA	Anandamide
ANOVA	One-way analysis of variance
BDNF	Brain-derived neurotrophic factor
C	Control
cAT-MSCs	Canine adipose tissue-derived mesenchymal stem cells
CB1	Cannabinoid receptor type 1
CB2	Cannabinoid receptor type 2
CBD	Cannabidiol
CD	Cluster of differentiation
CEUA	Ethics Committee on the Use of Animals
COX	Cyclooxygenase enzymes
D1	Dose 1
D2	Dose 2
DMEM	Dulbecco’s Modified Eagle’s Medium
DMSO	Dimethyl sulfoxide
eCBs	Endocannabinoids
ECS	Endocannabinoid system
EVs	Extracellular vesicles
FBS	Fetal bovine serum
GAPDH	Glyceraldehyde-3-phosphate dehydrogenase
GDNF	Glial cell line-derived neurotrophic factor
GM-CSF	Granulocyte-macrophage colony-stimulating factor
HGF	Hepatocyte growth factor
HPLC	High-performance liquid chromatography
HPRT	Hypoxanthine-guanine phosphoribosyltransferase
IFN-γ	Interferon-gamma
IL-2	Interleukin-2
IL-8	Interleukin-8
IL-10	Interleukin-10
LPS	Lipopolysaccharide
MSCs	Mesenchymal stem cells
MCP-1	Monocyte chemoattractant protein-1
PGE2	Prostaglandin E2
PPARγ	Peroxisome proliferator-activated receptor gamma
PTGES2	Prostaglandin E2 synthase 2
RPS5	Ribosomal protein S5
RPS19	Ribosomal protein S19
RT-qPCR	Real-time quantitative polymerase chain reaction
THC	Δ9-tetrahydrocannabinol
TRPV1	Transient receptor potential vanilloid 1
UNESP	São Paulo State University
